# Natural History of Asymptomatic Walled-off Necrosis in Patients With Acute Pancreatitis

**DOI:** 10.7759/cureus.34646

**Published:** 2023-02-05

**Authors:** Manish Kumar, Ujjwal Sonika, Sanjeev Sachdeva, Ashok Dalal, Poonam Narang, Bhawna Mahajan, Ankush Singhal, Siddharth Srivastava

**Affiliations:** 1 Gastroenterology, Govind Ballabh Pant Hospital, New Delhi, IND; 2 Radiology, Govind Ballabh Pant Hospital, New Delhi, IND; 3 Biochemistry, Govind Ballabh Pant Hospital, New Delhi, IND

**Keywords:** conservative management, walled-off necrosis, infection, asymptomatic, acute pancreatitis

## Abstract

Background and objectives

Studies on the natural history of asymptomatic walled-off necrosis (WON) in acute pancreatitis (AP) are scarce. We conducted a prospective observational study to look for the incidence of infection in WON.

Material and methods

In this study, we included 30 consecutive AP patients with asymptomatic WON. Their baseline clinical, laboratory, and radiological parameters were recorded and followed up for three months. Mann Whitney U test and unpaired t-tests were used for quantitative data and chi-square and Fisher’s exact tests were used for qualitative data analysis. A p-value <0.05 was considered significant. Receiver operating characteristic curve (ROC) analysis was done to identify the appropriate cutoffs for the significant variables.

Results

Of the 30 patients enrolled, 25 (83.3%) were males. Alcohol was the most common etiology. Eight patients (26.6%) developed an infection on follow-up. All were managed by drainage either percutaneously (n=4, 50%) or endoscopically (n=3, 37.5%). One patient required both. No patient required surgery and there was no mortality. Median baseline C-reactive protein (CRP) was higher in infection group 76 (IQR=34.8) mg/L vs asymptomatic group, 9.5 mg/dl (IQR=13.6), p<0.001. IL-6 and tumor necrosis factor (TNF)-alpha was also higher in the infection group. The size of the largest collection (157.50±33.59 mm vs 81.95±26.22 mm, P<0.001) and CT severity index (CTSI) (9.50±0.93 vs 7.82±1.37, p<0.01) were also higher in infection group as compared to the asymptomatic group. ROC curve analysis of baseline CRP (cutoff 49.5mg/dl), size of WON (cutoff 127mm) and CTSI (cutoff of 9) showed AUROC (area under ROC) of 1, 0.97, and 0.81 respectively for the future development of infection in WON.

Conclusion

Around one-fourth of asymptomatic WON patients developed an infection during three-months follow-up. Most patients with infected WON can be managed conservatively.

## Introduction

A majority of cases of acute pancreatitis are mild with uncomplicated course. However, in up to 20% of cases, pancreatic necrosis can be seen [1]. These patients can have mortality of 20-30% [2]. The term "walled-off necrosis" (WON) was first proposed in the year 2005 and later included in revised Atlanta Classification, 2012 [3,4]. WON occurs in 1-9% cases of acute pancreatitis and in 15% of severe acute pancreatitis. Approximately half of WON cases (37-59%) undergo spontaneous regression [5-8]. Symptomatic WON is an indication for treatment. But, there is no consensus on treatment of asymptomatic WON. Potential complications of asymptomatic WON include infection (up to 44%), rupture and GI bleed. Current guidelines recommend conservative management of asymptomatic WON, regardless of its size [9-12]. However, there is scant data to support these recommendations. The available studies are either retrospective with small sample size and short follow up or have heterogeneous study population [7-10,13,14].

We prospectively followed the patients with asymptomatic WON in acute pancreatitis to study the natural history and find out the variables that can indicate the need for drainage.

## Materials and methods

This is a prospective observational single-center study that included 30 consecutive asymptomatic patients with acute pancreatitis and WON from 1st November 2018 to 30th April 2020. Asymptomatic patients were those who were able to tolerate oral nutrition and had only mild, occasional abdominal pain or discomfort (not requiring analgesics regularly) without any infection, pressure symptoms, and GI bleeding. All patients were prospectively followed up monthly by OPD visits for a minimum of three months. If patients had new onset of symptoms such as pain, fever, vomiting, jaundice, or bleeding, they were admitted and managed accordingly. The presence of infection in WON was diagnosed by new onset/persistent organ failure (Marshall score ≥2 for each organ), new onset of systemic inflammatory response syndrome (SIRS) in the absence of other sources of infection, worsening clinical condition, the presence of air in the necrotic collection (on USG or CT), or gram stain/culture positive on necrotic material (if drained) [15-17].

Patients were divided into two groups (those who developed an infection during the follow-up and those who remained asymptomatic). Both the groups were compared based on their baseline clinical (including demographic), laboratory (biochemical and inflammatory markers), and radiological parameters. The inclusion criteria were ≥18 years <60 years of age and acute pancreatitis with asymptomatic WON. The exclusion criteria were comorbidities like diabetes mellitus, systemic hypertension, chronic obstructive pulmonary disease, chronic kidney disease, chronic liver disease, malignancy, chronic pancreatitis, post-endoscopic retrograde cholangiopancreatography (ERCP) pancreatitis, pregnancy, and HIV.

Outcome

The primary outcome was the development of infection, while secondary outcomes included the number and duration of hospitalizations, time duration for infection/drainage of WON (from the onset of pancreatitis), nutritional and sarcopenia assessment by BMI, hand grip dynamometry and psoas muscle index (PMI).

Study Protocol

Clinical data, anthropometry, hand grip dynamometry, and laboratory parameters were recorded at baseline. Routine biochemical parameters were done monthly for three months. CRP, IL-6, and TNF levels were done at baseline and at three months. All patients underwent contrast-enhanced CT abdomen (at baseline and further as decided by clinical condition, otherwise abdominal ultrasound was done monthly during the follow-up). Parameters included in the CT evaluation were psoas muscle mass index, pancreatic necrosis (modified CT severity index; CTSI), location and size of WON, the total number of collections, and the presence or absence of air in collections [18-19]. The largest dimension of the collection was taken as the size of WON. Data regarding organ failure and the bedside index for severity in acute pancreatitis (BISAP) score was taken at the time of admission at the onset of pancreatitis. Psoas muscle index (PMI) assessment was done by CT (NCCT cut) at L3/4 level as the cross-sectional area of B/L psoas muscle/height2 (cm2/m2). Low PMI is suggestive of low muscle volume (sarcopenia). Cutoff values taken for PMI (cm2/m2) were 3.89 and 3.20 for males and females respectively [19]. Hand grip strength was measured by a hand-held dynamometer. The mean of three measurements was taken. Appropriate age and sex-related cutoff values were taken [20].

Treatment Protocol

All patients who developed infected WON on follow-up were treated with broad-spectrum antibiotics (imipenem + cilastatin) and were sent for blood culture. CECT was done in all the patients. All patients with WON infection underwent drainage, either percutaneous or endoscopic. The decision for the mode of drainage was made based on imaging findings considering the distance of collection from the lumen, the thickness of the wall, the presence of collaterals, and debris. Endoscopic drainage was EUS guided, either transgastric or transduodenal approach, and a 30 mm x16 mm fully covered self-expandable metal stent (FC-SEMS) was placed. In case of no clinical improvement within 72 hours, an endoscopic necrosectomy was performed. The SEMS was removed within four weeks of the procedure after confirmation of the resolution of the collection on USG. Percutaneous catheter drainage (PCD) was done with a 12 Fr drain under USG guidance. More than one drain was used in multiple collections. The preferred route was through the retroperitoneum. PCD was upgraded to 14/16 Fr in cases of inability to drain the thick collection. Drains were removed when there was no residual drainable collection left. During both modes of drainage, the first sample was sent for culture, and antibiotics were tailored as per the sensitivity pattern.

Statistical Analysis

Differences in quantitative data between the two groups were tested by Student’s t-test (unpaired) or Mann-Whitney U test. Differences between the proportions were tested by the chi-square test or Fisher’s exact test. A p-value of less than 0.05 was considered statistically significant.

## Results

**Figure 1:**
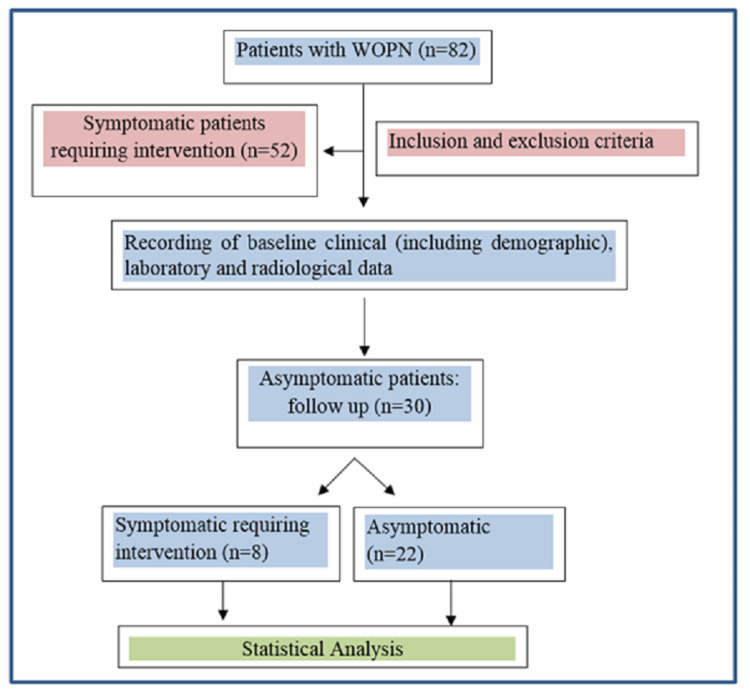
Study protocol of patients with WOPN (Walled off pancreatic necrosis)

A total of 30 patients were enrolled (Figure 1); 25 (83.3%) were males and five were females (16.7%), with a mean age of 30.57±9.25 years. Alcohol consumption was the most common etiology, seen in 16 (53.3%) patients followed by biliary in five (16.7%) patients. Eight (26.7%) patients developed an infection of WON during the follow-up, after a mean duration of 72.48±16.3 days from the onset of acute pancreatitis (AP). New-onset symptoms were fever in six (75%) patients and unremitting abdominal pain in five (62.5%) patients. New onset SIRS was present in six (75%) patients. The rest of the 22 (73.3%) patients remained asymptomatic during the follow-up period of three months. Six patients had positive growth on the pus culture.

Percutaneous catheter drainage (PCD) was done in four (50%) patients, transluminal drainage using fully covered SEMS (FC-SEMS) in three (37.5%) and FC-SEMS followed by PCD in one (12.5%) patient; they experienced failure of resolution of WON following SEMS and subsequently, PCD was done. Only one patient required endoscopic necrosectomy while upgradation of PCD was required in three cases.

There was no complication to drainage in any patient except for external SEMS migration (into stomach) in one patient, which was successfully removed endoscopically. In all eight patients, there was a complete resolution of WON within a mean duration of 35.25±8.53 days. SEMS was removed within four weeks in all the patients. The mean duration of hospitalization was 18.62±7.7 days. None of the patients required surgery and there was no mortality. WON culture showed growth of Pseudomonas aeruginosa in three patients; Klebsiella pneumoniae, Enterococcus, and E. coli; each was isolated in one patient.

In the asymptomatic group, 14/22 (63.6%) patients showed regression of mean WON size from 76.57±21 to 51.07±21.62 mm over three months. There was no change of size in the rest of the eight (36.3%) patients.

Comparison of clinical (including demographic), laboratory, and radiological variables between infected and asymptomatic patients

Clinical (Including Demographic) Variables

The mean age was similar. Though the frequency of alcohol consumption was 81.2% (13 patients) in the asymptomatic group vs. 18.8% (three patients) in the infection group, this difference was not statistically significant, p=0.56. BISAP score was significantly higher in the infection group, 1.63±0.74 vs. 0.64±0.58 in the asymptomatic group, p=<0.01 (Table 1).

**Table 1:** Comparison of clinical and demographic variables in subjects with and without WON infection WON: walled-off necrosis; BISAP: bedside index for severity in acute pancreatitis

Variable	Patients asymptomatic during follow-up (n=22)	Patients with WON infection (n=8)	P-value
Male: Female	17:5	8:0	0.28
Age in years	28.45±7.55	36.38±11.45	0.03
BMI, Kg/m2	22.71±1.62	24.64±2.59	0.09
Etiology			
Alcohol consumption	13 (59.09%)	3 (37.5%)	0.56
Gall stones	3 (13.63%)	2 (25%)	
Others	6 (27.27%)	3 (37.5%)	
BISAP score			
0	9 (40.9%)	0 (0.0%)	0.01
1	12 (54.54%)	4 (50%)	
2	1 (4.54%)	3 (37.5%)	
3	0 (0.0%)	1 (12.5%)	
BISAP score (at the onset of pancreatitis)	0.64±0.58	1.63±0.74	<0.01
Transient organ failure (at the onset of pancreatitis)			
No	20 (90.9%)	5 (62.5%)	0.10
Yes	2 (9.09%)	3 (37.5%)	
Persistent organ failure (at the onset of pancreatitis)			
No	22 (100.0%)	5 (62.5%)	0.10
Yes	0	3 (37.5%)	
Hand grip strength (dominant hand)			
Low	5 (22.72%)	2 (25%)	1.0
Normal	17 (77.27%)	6 (75%)	
Hand grip strength (non-dominant hand)			
Low	8 (36.36%)	4 (50%)	0.68
Normal	14 (63.63%)	4 (50%)	

Laboratory (Biochemical, Hematological, and Inflammatory) Variables

**Figure 2:**
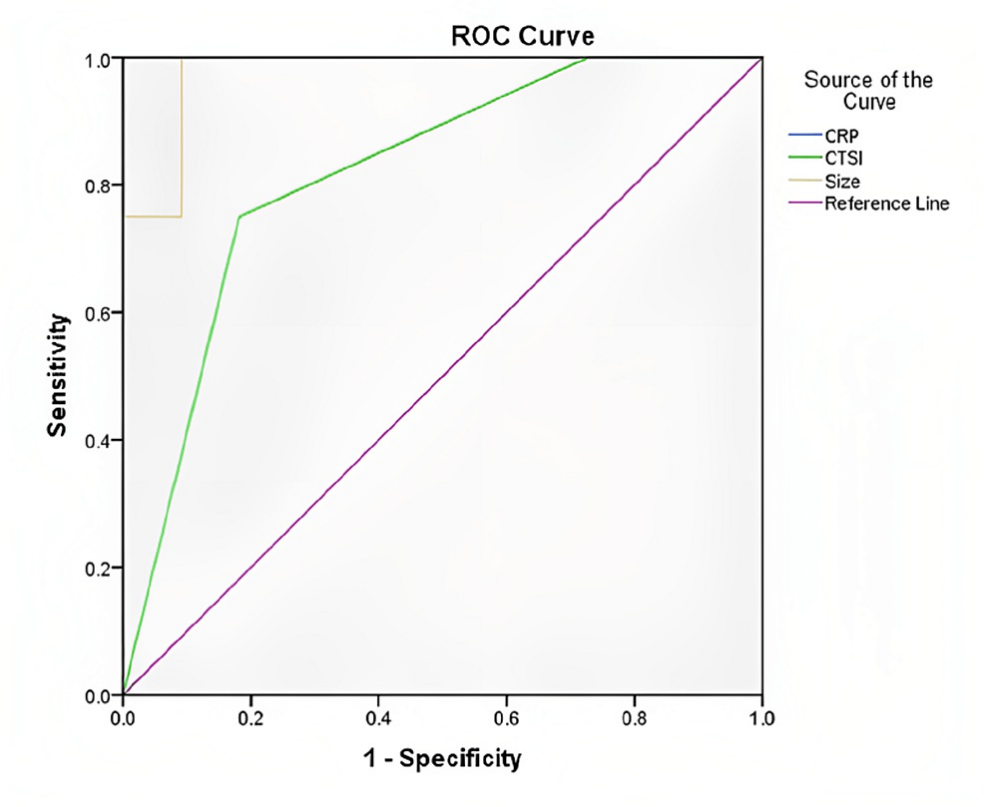
ROC curve for the relationship between CRP, modified CSTI as well as size and infection of WON ROC: Receiver operating characteristic; CRP: C-reactive protein; CSTI: CT severity index; WON: walled-off necrosis

There was no difference in routine biochemical and hematological parameters between the two groups. Median baseline CRP levels in the infection group were 76 (56.2-91) mg/L vs 9.5 (3-16.6) mg/L in the asymptomatic group, p<0.001. ROC curve of baseline CRP and infection in WON had an area under curve (AUROC) of 1, with a sensitivity of 87.5% and specificity of 100% at a cutoff of 49.5 mg/L (Table 2). Both positive and negative predictive value at this cutoff is 100% each (Figure 2) 

**Table 2:** Comparison of laboratory (biochemical and hematological) variables in subjects with and without WON infection TLC: total leucocyte count; AST: aspartate transaminase; ALT: alanine transaminase; ALP: alkaline phosphatase; INR: international normalized ratio; RBS: random blood sugar; CRP: c-reactive protein; WON: walled-off necrosis

Variable	Patients asymptomatic on follow-up (n=22)	Patients with WON infection (n=8)	P value
Hematological			
Hemoglobin (g/dL)	10.29±1.87	11.32±3.40	0.55
TLC (cells/mm^3^)	8218.18±2945.32	10975.0±4931.46	0.12
Platelets (lacs/mm^3^)	2.26±1.13	2.17±0.94	0.96
Biochemical			
Bilirubin (mg/dL)	0.80±0.34	0.69±0.27	0.32
AST (IU/L)	39.09±14.23	35.38±14.22	0.58
ALT (IU/L)	34.05±16.93	29.13±9.22	0.57
ALP (IU/L)	139.36±34.91	142.38±36.38	0.57
S. total protein (g/dL)	7.12±0.66	7.12±0.89	0.79
S. albumin (g/dL)	3.43±0.53	3.66±0.99	0.67
INR	1.08±0.13	1.11±0.17	0.59
Creatinine (mg/dL)	0.74±0.22	0.86±0.25	0.34
RBS (mg/dL)	111.82±29.09	124.13±38.29	0.52
CRP (mg/L)	9.5 (3-16.6)	76 (56.2-91)	<0.001
Calcium (mg/dL)	8.40±0.52	8.41±0.79	0.81
IL-6 (pg/ml)	48.50 (10.58-62.88)	350 (252.5-400)	<0.001
TNF-α (pg/ml)	8 (8-9)	15 (8.12-130.25)	0.03

Median IL-6 levels in the infection group and the asymptomatic group were 350 (252.5-400) pg/ml and 48.50 (10.58-62.88) pg/ml, respectively, p<0.001. TNF-α levels also showed a statistically significant difference between the two groups, with median TNF-α levels in the infection group and asymptomatic group of 15 (8.12-130.25) pg/ml and 8 (8-9) pg/ml, respectively with a p-value of 0.03.

Radiological Variables

The mean size of WON was 157.5±33.59 mm in the infection group and 81.95±26.22 mm in the asymptomatic group, P<0.001. The size of WON in patients developing infection changed from 157.5±33.59 mm at baseline to 151.1±35.4 mm before drainage. The size remained the same in one, decreased in five, and increased in two patients. The ROC curve between size and infection in WON had an AUROC of 0.97, with a sensitivity of 87.5% and specificity of 91% at the cutoff of 127 mm. The positive predictive value (PPV) at this cutoff is 87.5% and the negative predictive value (NPV) is 100% (Figure 2) (Table 3).

**Table 3:** Comparison of radiological variables in subjects with and without WON infection (on CT abdomen) WON: walled-off necrosis; CSTI: CT severity index

Variable	Patients asymptomatic during follow-up (n=22)	Patients with WON infection (n=8)	P value
Psoas muscle index, cm^2^/m^2^			
Low	5 (71.42%)	2 (28.57%)	0.71
Normal	17 (73.91%)	6 (26.08%)	
Necrosis of pancreas			
≤30%	16 (94.1%)	1 (5.9%)	<0.01
>30%	6 (46.2%)	7 (53.8%)	
Size of WON, mm	81.95±26.22	157.50±33.59	<0.001
Modified CTSI	7.82±1.37	9.50±0.93	<0.01
No. of collections			
1	13	6	0.57
2	7	1	
≥3	2	1	
Location of collection			
Head	3	1	1.0
Body	20	8	0.99
Tail	16	8	0.15

Modified CT severity index (CTSI) was 9.5±0.93 in the infection group vs. 7.82±1.37 in the asymptomatic group, p <0.01. The ROC curve between CTSI and infection in WON showed an AUROC of 0.81, with a sensitivity of 75% and specificity of 81% at a CTSI cutoff of 9. PPV at this cutoff is 60% and NPV 90% (Figure 2).

## Discussion

We found that over a period of three months, most of the patients with asymptomatic WON (n=22, 73.3%) did not develop any symptoms and did not require intervention. Only 26.7% (n=8) of patients developed an infection and all of them could be managed without surgical intervention with no mortality.

Studies on follow-up of asymptomatic patients with WON showed that >50% have either decreased in size or resolution of collection without any intervention [9,21]. In our study, 14 (46.6%) patients had regression of WON size, from 76.57±21 mm to 51.07±21.62 mm during the three-month follow-up. This supports the current recommendation of conservative management in these patients, regardless of WON size. The other side of conservative management is the development of complications in 30-56% of patients, infection being the most common, seen in 9-44% of patients. According to the published literature complications usually occur in the first few months, usually within 1.63-3.5 months [9,10,21]. In this study too, all the complications developed within 72.48±16.3 days. This highlights the importance of close monitoring, especially for the initial months. The majority of the complications could be successfully treated with minimally invasive techniques, without any mortality [9,10,21]. In our study, all eight patients were successfully managed by minimally invasive techniques (PCD or endoscopic drainage).

Our study is probably the first study to use inflammatory markers like IL-6 and TNF-α in the study of asymptomatic WON cases. Both these markers were significantly elevated in the infection group vs. the asymptomatic group in our study. The use of inflammatory cytokines like TNF-α and IL-6 might not be feasible in all the centers, due to the cost and accessibility constraints. However, CRP can be used, having the advantage of low cost and easy availability. The use of CRP as a predictor of complication has also been shown by Wroński et al [10]. Among all the parameters that were significant on comparison between the two groups, AUROC was the highest for CRP, with a value of 1 (Figure 2). Based on ROC, a CRP level cutoff of 49.5 mg/L can help in decision-making regarding drainage of the WON (Figure 2).

Regarding the size of WON, the literature shows variable results. While Rana et al. showed that the size of WON was not significantly higher in patients who developed complications, Jagielski et al. showed that patients with complications had larger WON size, 121.9±25.9 mm vs. patients with regression of WON, 90.8±25.6 mm, p<0.01 [9,21]. Our study showed results similar to the latter study. The reason for this difference could be because of the larger WON size in our study and the study by Jagielski et al., 102.1±43.8 mm and 100.21 ± 25.7 mm, respectively vs. 82±22 mm in the study by Rana et al [9,21].

Contrary to the study by Wroński et al., which has not shown any significant differences with respect to CTSI, our study showed higher CTSI in the infection group, 9.50±0.93 versus 7.82±1.37 in the asymptomatic group, p=<0.01 [10]. This may be explained, at least partly by the significantly higher number of patients with pancreatic necrosis (>30%) in the infection group, 53.8% vs. 5.9% in the asymptomatic group, p=<0.01.

Among all the radiological parameters that were significant on comparison, AUROC was highest for the size of WON (AUROC of 0.97) with 87.5% sensitivity and 91% specificity for WON infection at a size cutoff of 127 mm. CTSI had AUROC of 0.81, and at a cutoff of 9 has a sensitivity of 75% and specificity of 81%. When CTSI and the size of WON are taken together, both can help in decision-making about the drainage of collection, and CECT abdomen can be utilized as the most useful modality for guiding, keeping in mind its easy availability and cost.

The main limitation of our study is the small sample size. Our institute being a referral center, the majority of the patients that were referred were symptomatic and therefore excluded. Hence, we could only enroll 30 asymptomatic WON patients. The high predictive values of parameters eg. CRP etc. should be validated in another cohort of patients, but because of a small number of patients with asymptomatic WON, we could not have a validation cohort.

The strength of our study is the prospective study design and a homogenous study cohort of acute pancreatitis patients with asymptomatic WON as there are few studies available that have evaluated the natural history of asymptomatic walled-off necrosis. The available studies are retrospective with a heterogeneous study population. To the best of our knowledge, this is probably the first study that has prospectively followed patients with asymptomatic walled-off necrosis and aimed to find out the variables that can predict WON infection in these patients. Also probably this is the first study to use inflammatory markers like IL-6 and TNF-α in the study of asymptomatic WON cases.

## Conclusions

Conservative management of patients with asymptomatic WON is a safe strategy as only around one-fourth of patients in our study developed an infection during the three-month follow-up. All such patients could be managed by minimally invasive techniques, without any mortality. CECT abdomen and CRP can be utilized to guide the need for intervention in these patients. Further prospective studies with larger sample sizes are needed to confirm these observations.
